# Supplementary material for: Leber Hereditary Optic Neuropathy: Molecular Pathophysiology and Updates on Gene Therapy
Source: Biomedicines. 2022 Aug 9;10(8):1930. doi: 10.3390/biomedicines10081930 (PMC9405679; doi:10.3390/biomedicines10081930)
Supplement: Supplementary file 1 [file biomedicines-10-01930-s001.zip › biomedicines-1809316-supplementary.pdf]

Recent advance in gene therapy for Leber hereditary optic neuropathy: a systematic review

(Supplemental Materials)

Supplemental Material

Supplemental Material S1. Search strategy

Supplemental Material S2. Assessment of Cochrane Risk of Bias tool (ROB)

Supplemental Material S3. Assessment of Newcastle-Ottawa Scale (NOS) tool

Supplemental Material S1

Search strategy

Supplemental Material S1
Search strategy

Primary search steps:
1. Leber hereditary optic neuropathy
2. Leber hereditary optic atrophy
3. Leber optic atrophy
4. LHON
5. gene therapy
6. recombinant gene
7. gene delivery
8. (#1 or #2 or #3 or #4) and (#5 or #6)

Final syntax in PubMed (an example):
((((Leber hereditary optic neuropathy) OR (Leber hereditary optic atrophy)) OR (Leber optic atrophy)) OR (LHON)) AND (((gene therapy) OR (recombinant gene)) OR (gene delivery)) Sort by: Publication Date

Supplemental Material S2

Assessment of Cochrane Risk of Bias tool (ROB)

Supplemental Material S2

Assessment of Cochrane Risk of Bias tool (ROB)

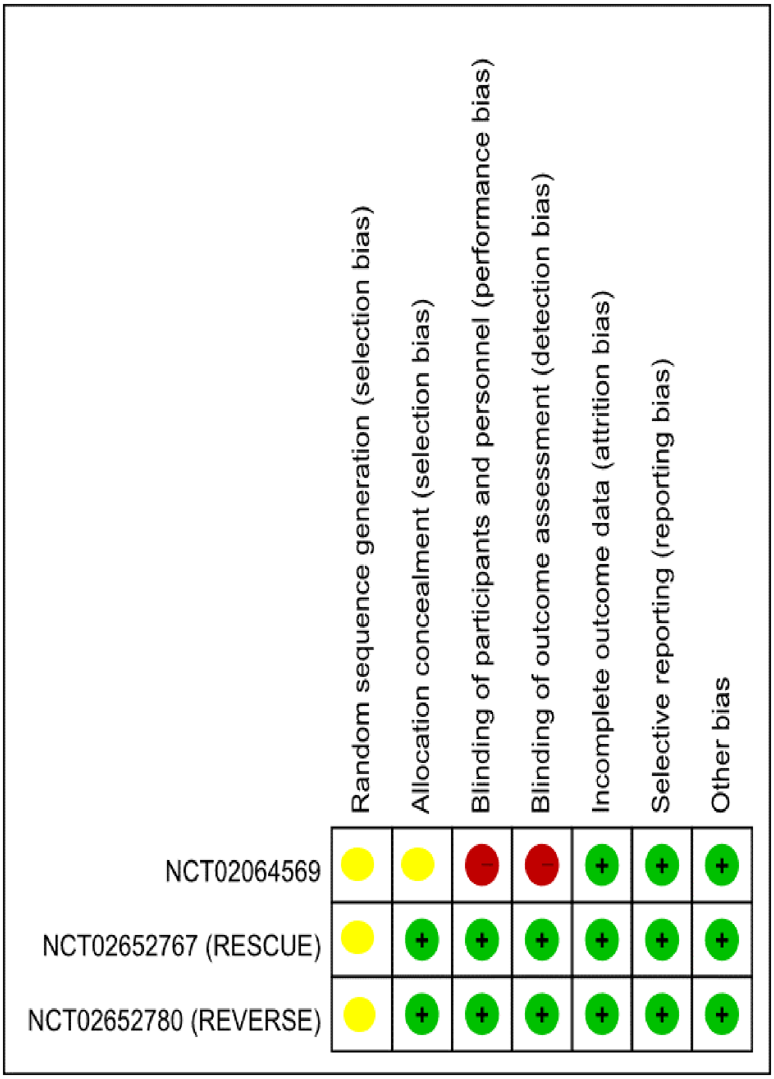

Supplemental Material S3

Assessment of Newcastle-Ottawa Scale (NOS) tool

Supplemental Material S3

Assessment of Newcastle-Ottawa Scale (NOS) tool

Trials	Selection				Comparability	outcomes			Total quality score
	Representativeness of the exposed cohort	Selection of the non-exposed cohort	Ascertainment of exposure	Demonstration that outcome of interest was not present at start of study	Comparability based on the design or analysis	Assessment of outcome	Was follow-up long enough for outcomes to occur	Adequacy of follow up of cohorts	
**NCT02161380**	*	*	*		*	*		*	6
**NCT03153293**	*	*	*		*	*		*	6
**NCT03406104**	*	*	*		**	*	*	*	7
**EUDRACT N° 2013-001405-90.**	*	*	*		**	*	*	*	7
